# Uncommon cells in cerebrospinal fluid in Japanese encephalitis: A case report

**DOI:** 10.1097/MD.0000000000043492

**Published:** 2025-08-29

**Authors:** Yuting Hou, Xiao Yang

**Affiliations:** a Department of Cerebrospinal Fluid Laboratory, The General Hospital of Ningxia Medical University, Yinchuan, Ningxia, China; b The First Clinical Medical College of Ningxia Medical University, Ningxia Medical University, Yinchuan, Ningxia, China; c Department of Neurology, The General Hospital of Ningxia Medical University, Yinchuan, Ningxia, China.

**Keywords:** cerebrospinal fluid, cytomorphology, cytopathology of infectious diseases, diagnosis, Japanese encephalitis

## Abstract

**Rationale:**

Cerebrospinal fluid (CSF) cytomorphology plays a critical role in the diagnosis of central nervous system infections. During an outbreak of Japanese encephalitis (JE) in Ningxia, China, an uncommon CSF cytomorphology in 1 case posed diagnostic challenges, highlighting the need to correlate cellular findings with clinical context for accurate interpretation.

**Patient concerns:**

A 56-year-old female farmer from a JE-endemic village presented with fever, headache, dizziness, and nausea. Initial CSF cytological analysis revealed 2 uncommon cell types: type I cells, resembling siderophages, and type II cells mimicking malignant tumor cells.

**Diagnoses:**

JE was confirmed through JE virus-specific immunoglobulin G antibodies in both serum and CSF and genotype Ib identification. No evidence of cerebral hemorrhage or neoplastic meningitis was found. These 2 cell types disappeared during subsequent follow-up examinations.

**Interventions:**

Intravenous ganciclovir (300 mg, Q12H) was initiated promptly.

**Outcomes:**

Symptoms resolved within 1 week. At the 1-month follow-up, the patient recovered completely, confirming the JE diagnosis.

**Lessons:**

Transient uncommon CSF cells in this case of JE may exhibit morphological features resembling siderophages or malignant tumor cells, thus requiring a comprehensive correlation between cytomorphological findings and both clinical and laboratory data. This case highlights CSF cytology’s diagnostic value in central nervous system infections while cautioning against overreliance on isolated cellular findings. Effective collaboration between clinicians and cytologists is essential to prevent misdiagnosis and ensure appropriate therapeutic guidance.

## 1. Introduction

The cytomorphology of cerebrospinal fluid (CSF) is an essential component of the neurologist’s diagnostic workup and represents a rapid and cost-effective screening method for various central nervous system (CNS) disorders.^[1]^ Viral meningitis is typically characterized by a predominance of lymphocytes in the CSF,^[2]^ as well as the presence of activated lymphocytes and plasma cells.^[1,3,4]^ Japanese encephalitis (JE) is an acute CNS disease caused by the Japanese encephalitis virus (JEV), which is transmitted via mosquito vectors.^[5]^ An epidemic outbreak of JE was reported in Ningxia, located in the northwest region of China,^[6,7]^ during which a remarkable case with distinctive CSF cytological features drew our attention. Although the patient was eventually diagnosed with JEV infection, the presence of 2 uncommon types of cellular components in the initial lumbar puncture led to diagnostic challenges. There is a paucity of case reports and limited photographic illustrations documenting these 2 cell types in the existing literature. We present this case to emphasize the importance of comprehending the morphological features of immune cells and benign components in CSF, as the CSF cytomorphology is an invaluable diagnostic window for CNS disorders.

## 2. Case report

A 56-year-old female farmer presented with symptoms of fever, diaphoresis, and chills. Two days later, she developed a headache, dizziness, and nausea, with a body temperature of 39.6°C. She exhibited mild expressive aphasia but with preserved comprehension abilities. There were no reports of vomiting, coughing, syncope, limb weakness, numbness, or urinary incontinence. She was initially taken to a local hospital where the cranial computed tomography scan revealed no abnormalities. Due to persistent symptoms, she was subsequently transferred to our hospital.

On admission, she had a body temperature of 40°C, a pulse rate of 124 beats/min, a blood pressure of 125/75 mm Hg, and a respiration rate of 22 breaths/min. Neurological tests revealed no abnormalities. Routine laboratory tests showed a slightly elevated monocyte count of 0.83 × 10^9^/L in the peripheral blood (upper normal limit, 0.60 × 10^9^/L), a fibrinogen level of 4.59 g/L (upper normal limit, 4.00 g/L), and an activated partial prothrombin time of 20.7 seconds (normal range, 23–35 seconds).

Serological tests for herpes simplex virus, rubella virus, cytomegalovirus, toxoplasma, brucella, syphilis, and human immunodeficiency virus, as well as blood cultures, yielded negative results. Hematological tests for autoimmune diseases, including immunoglobulin G, immunoglobulin A, immunoglobulin M, rheumatoid factor, complement C3, complement C4, anti-ribonucleoprotein antibody, anti-Sjögren syndrome antigen A antibody, anti-Sjögren syndrome antigen B antibody, anti-Scl-70 antibody, anti-Jo-1 antibody, anti-Sm antibody, and anti-ribosomal P protein antibody, were all negative. However, the anti-nucleus antibody-HEp-2 test was positive at a titer of 1:100. Enhanced brain magnetic resonance imaging revealed nonspecific multiple patchy ischemic demyelination in the anterior and posterior horns bilaterally, as well as in the half-oval center. An infectious disease screening of the CNS using CSF, including alcian blue staining, acid fast staining, and microbial culture, were all negative.

The initial lumbar puncture revealed an opening pressure of 140 mm H_2_0 and CSF white blood cell count of 27 × 10^6^/L. The protein content was 0.28 g/L, glucose was 3.7 mmol/L, and chloride was 121 mmol/L, all within the reference range (Table 1, day 0). Five drops of the CSF were pipetted into a cytofunnel and centrifuged at 113g for 5 minutes at room temperature. The air-dried slides were stained using May-Grunwald–Giemsa staining. The smear showed 61% lymphocytes, 24% monocytes, 8% neutrophils, and 7% uncommon cells.

**Table 1:** The results of CSF biochemical tests and cytomorphology.

Hospital day	0	8	11	16	Reference intervals
Color of the supernatant	Crystal clear	Crystal clear	Crystal clear	Crystal clear	Crystal clear
CSF opening pressure	140	120	130	130	60–200 mm H_2_O
CSF-protein	0.28	0.43	0.22	0.40	0.12–0.60 g/L
CSF-glucose	3.7	3.0	2.9	2.9	2.2–3.9 mmol/L
CSF-chloride	121	125	125	123	120–132 mmol/L
White blood cell counts	27	10	10	2	<5×10^6^/L
Red blood cell counts	200	100	–	–	0 × 10^6^/L
Lymphocytes (%)	61	95	96	–	Approximately 70%
Monocytes (%)	24	2	2	–	Approximately 30%
Neutrophils (%)	8	3	2	–	Occasionally appeared
Uncommon cells (%)	7	–	–	–	–

CSF = cerebrospinal fluid.

**Figure 1.**
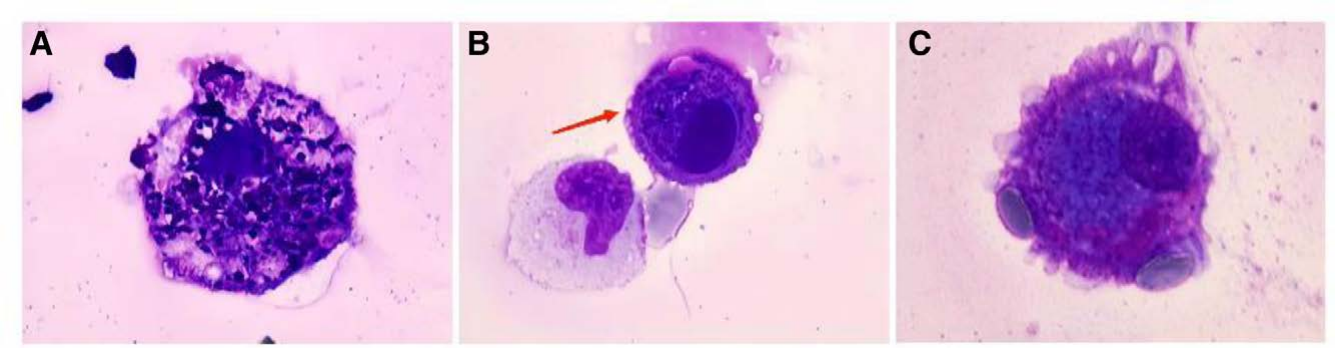
Morphological features of 2 uncommon types of cells in CSF on May-Grunwald–Giemsa staining (×1000). (A) The type I cells was obviously enlarged in volume, with strong basophilic staining, contained numerous, variably sized, purple or blue inclusions surrounded by vacuoles. (B) The type II cell (arrow) had abundant cytoplasm, with the central part of the cytoplasm stained dark blue and the edges appeared pink–magenta. The cell exhibited eccentric nucleus, fine nuclear chromatin, and inconspicuous nucleolus. Small protrusions of cytoplasm were dissected from the cell. In the lower left region of the cell, there were a red blood cell and a monocyte. (C) Another type II cell was enlarged in volume, with abundant dense pink–magenta cytoplasm, nucleus located on the edge of the cytoplasm, and displayed irregular cytoplasmic protrusions and surface blebs. Next to the uncommon cell were 2 red blood cells. [The color figures were acquired using a ProgRes^®^ SpeedXTcore3 camera and ProgRes^®^ CapturePro software version 2.8.8 (Jenoptik, Germany).] CSF = cerebrospinal fluid.

The type I cells exhibited obvious enlargement in volume, with strong basophilic staining, contained numerous, variably sized, coarse granular inclusions that appeared purple or blue, and were surrounded by vacuoles (as shown in Fig. 1A). Another, the type II cells exhibited a slight increase in volume with eccentrically positioned and hyperchromatic nucleus. The cytoplasm was abundant and stained dark blue, while the cell edges appeared pink–magenta. Irregular cytoplasmic protrusions and surface blebs were also observed. However, these cells maintained regularly shaped nucleus and a normal nuclear-to-cytoplasmic ratio (as shown in Fig. 1B, C).

Although the type II cells resembled malignant tumor cells, the disease onset coincided with the outbreak of JE, and she came from a village with the highest number of JE cases.^[6,7]^ The patient presented with acute headache and fever. The cytomorphology of CSF revealed lymphocyte predominance, while CSF biochemical tests showed normal results. Overall, her epidemiological history, clinical presentation, and CSF test findings were consistent with viral infection of the CNS.

We prioritized the suspicion of JEV infection and empirically administered ganciclovir antiviral therapy (300 mg, Q12H), followed by laboratory tests confirming the presence of immunoglobulin G anti-JEV antibodies in both serum and CSF samples. Subsequently, the JEV isolated from this patient’s stored serum and CSF, as well as other JE cases, were identified as belonging to genotype Ib.^[7]^ Simultaneously, we tested a wide range of serum tumor markers, including carbohydrate antigen (CA) 125 (7.12 U/mL), CA153 (17.8 U/mL), CA199 (10.39 U/mL), carcinoembryonic antigen (1.42 ng/mL), alpha-fetoprotein (1.59 ng/mL), and neuron-specific enolase (8.54 ng/mL), which were all within the normal range. The CSF was sampled three times during treatment, with the results shown in Table 1 indicating the eventual normalization of CSF white blood cell. These 2 uncommon cell types were not observed subsequently. Meanwhile, close monitoring of the patient’s condition revealed a rapid improvement in symptoms over the following days. Thirteen days after admission, the patient demonstrated recovery and was subsequently discharged. At a 1-month follow-up appointment, she had achieved complete recovery, thereby confirming the initial diagnosis of Japanese encephalitis.

## 3. Discussion

Initially, the morphology of the type I cells raised concern about potential siderophages. Bleedings within the CSF compartment induce macrophages activation and phagocytosis of erythrocytes, which then are called erythrophages.^[8]^ Erythrophages are considered to occur 12 to 18 hours after the bleeding event.^[9,10]^ After an additional 36 to 48 hours, erythrophages produce haemosiderin deposits and are then called siderophages.^[9,10]^ Abundant siderophages persist for days to weeks after the bleeding event. Of note, single siderophage may be encountered upon repeated lumbar punctures as a sign of minor traumatic taps.^[1]^ The siderophage displays foamy cytoplasm with bluish hemosiderin particles.^[1]^ In this case, the patient’s physical examination findings, clinical presentations, and CSF parameters made a serious diagnosis unlikely. Prior to this hospitalization, no lumbar puncture had been performed.

We identified the type I cells as activated monocytes, which are known to infiltrate the CNS during inflammatory processes.^[11]^ JEV infection in the human brain directly damages neurons.^[12]^ The activated monocytes differentiate into macrophages and perform phagocytosis and cytokine secretion, leading to changes in cell morphology characterized by the enlarged volume, the broadened cytoplasm with vacuoles containing coarse granules.^[1]^ The morphology of the type I cells aligned with the aforementioned descriptions. Interestingly, the cytograms of the type I cells resembled those of macrophages described in patients with 5q-associated spinal muscular atrophy treated with nusinersen.^[13–15]^

After observing the cellular features of the type II cells, the malignant tumor cells were considered to be the primary possibility. The type II cells exhibited certain morphological features commonly found in malignant cells.^[1,16]^ The cell volume was significantly increased, the nucleus became hyperchromatic, and the cytoplasm became basophilic, exhibiting irregular cytoplasmic protrusions and surface blebs. In contrast, the type II cells had regularly shaped nucleus and maintained normal nuclear-to-cytoplasmic ratio, suggesting benign features.

The headache associated with neoplastic meningitis (NM) progressively worsens and becomes barely tolerable, showing no response to conventional analgesic treatment. It exhibits distressing characteristics similar to those of a malignant disease, often accompanied by nausea and vomiting. Some patients may experience epileptic seizures, mental abnormalities, and impaired consciousness. The CSF features of NM include increased opening pressure, low levels of CSF-glucose, and elevated levels of CSF-protein.^[17]^ In this case study, there was no documented history of a prior cancer diagnosis. The CSF opening pressure was found to be within normal limits, with a predominance of lymphocytes. Additionally, the glucose and protein levels were observed to remain within the reference ranges. Following antiviral management, her symptoms exhibited a rapid improvement. Overall, given the detailed patient history, clinical presentations, imaging data, and CSF cytology results, as well as the drug treatment response – none of which provided evidence supporting NM – the inclination leant towards viral meningitis.

The slides were reviewed, and we identified the type II cells as normal ependymal cells. The ependymal cells in the CSF are enlarged, with abundant cytoplasm, round to oval nuclei, fine chromatin, and inconspicuous nucleoli. These cells exhibit prominent basophilic staining and possess ciliated surfaces. They are slightly larger than lymphocytes but maintain uniformity in terms of size, shape, and appearance, and occur most frequently in clusters.^[18–21]^ Ependymal cells are rarely found in the CSF and are considered to be of little diagnostic value. They can be seen in normal CSF samples as part of a traumatic artifact from the lumbar puncture needle, as well as in hydrocephalic children, CNS infections, subarachnoid hemorrhage, surgical interventions, and ischemic infarcts.^[19–21]^ We hypothesized that denudation may represent a primary response to ependymal injury, wherein ependymal cells are shed into the CSF during the acute phase of the disease and are subsequently phagocytosed and degraded by monocytes, macrophages, or other relevant cell types.

## 4. Conclusions

This case enhances our understanding of the morphological features of immune cells and benign components, which may potentially contribute to false positive diagnoses in the context of hemorrhage and malignancy. It further emphasizes the importance of cautioning against overreliance on isolated cellular findings. The identification of these 2 cell types in the CSF necessitates a comprehensive assessment of the patient’s clinical status, encompassing epidemiological history, clinical presentations, imaging studies, laboratory data, and the response to drug treatment. Effective collaboration between clinicians and cytologists is crucial to prevent misdiagnosis and ensure appropriate therapeutic guidance.

## Author contributions

**Conceptualization:** Yuting Hou, Xiao Yang.

**Data curation:** Yuting Hou, Xiao Yang.

**Investigation:** Yuting Hou, Xiao Yang.

**Methodology:** Yuting Hou, Xiao Yang.

**Project administration:** Yuting Hou.

**Writing – original draft:** Yuting Hou, Xiao Yang.

**Writing – review & editing:** Yuting Hou.
